# Urothelial Carcinoma: Role of Biomarkers in Diagnosis, Prognosis and Treatment

**DOI:** 10.3390/biomedicines13071696

**Published:** 2025-07-11

**Authors:** Ioannis Zachos

**Affiliations:** Department of Urology, University Hospital of Larisa, University of Thessaly, 41335 Larisa, Greece; johnbzac@yahoo.gr

## 1. Introduction

Urothelial carcinoma (UC), also known as transitional cell carcinoma (TCC), is the second most frequently diagnosed urological tumor worldwide, and typically involves the bladder. However, it can also manifest in the ureters, renal pelvis, and urethra. Approximately 70–75% of cases are non-invasive or low-grade, and the key to treatment is early detection [1]. However, the gold standard methods for diagnosis are cystoscopy or ureteroscopy for the upper tract; both approaches have disadvantages due to their invasiveness and cost. This has prompted research into identifying biological markers for the diagnosis, prognosis, and treatment of UC, thereby filling the gap in the aforementioned standard methods of evaluation [2]. The search for reliable, non-invasive urinary biomarkers has been ongoing for decades, driven by the potential to enhance early detection, risk assessment, and monitoring while minimizing dependence on cystoscopy and cytology [3,4]. Current urinary biomarkers, including assays like NMP22 and BTA, as well as DNA and RNA tests such as UroVysion, FISH, and Cxbladder, show higher sensitivity than cytology for detecting high-grade or recurrent disease. However, this increased sensitivity often comes with reduced specificity, especially in benign conditions. Although several markers are FDA-approved and commercially available, their clinical utility remains largely adjunctive [5,6,7]. The purpose of this Special Issue, “Urothelial carcinoma: The Role of biomarkers in diagnosis, prognosis, and treatment,” was to collect insightful articles that illuminate the growing role of biomarkers, ranging from diagnostic assays that enable non-invasive detection to molecular profiles that aid in prognosis and guide treatment decisions.

Across these contributions, we see a diverse array of innovations, highlighting the complexity of the disease and the potential for biomarker-informed strategies. The increasing understanding of genomic alterations, expression patterns, and microenvironmental factors promises to enhance patient outcomes and inform the design of more effective and less toxic therapeutic interventions. On this matter, this Editorial aims to both elaborate on each contributing text and inspire the reader to explore them.

## 2. An Overview of the Published Articles

Alexei Croitor’s article (contribution 1) aimed to analyze the clinical and pathological features and outcomes of bladder cancer in patients aged 18–45. They retrospectively analyzed 60 patients aged 18–45 who underwent transurethral resection of bladder tumor (TURBT) over 9 years. They found that 80% of tumors were non-muscle-invasive, with 16.7% being papillary urothelial neoplasms of low malignant potential (PUNLMP) and 43.3% being high-grade tumors. Recurrence was observed in 40% of the patients, while progression was noted in 16.7%. The overall survival rate at three years was 93.3%, and the progression-free survival rate was 83.3%. The study concludes that young patients predominantly present with low-to-intermediate-stage tumors; however, a notable proportion displays high-grade tumors correlated with poorer outcomes. These findings indicate that while bladder cancer in younger patients is often less aggressive, close follow-up and treatment are essential for those with high-grade tumors.

The article by Sara Meireles et al. (contribution 2) is focused on molecular profiling in upper tract urothelial carcinoma (UTUC) with synchronous or metachronous urothelial bladder cancer (UBC). The research analyzed the immunohistochemical (IHC) and genetic differences between UTUC-only and UTUC with synchronous or metachronous upper urinary tract urothelial carcinoma (UTUC + UBC). It evaluated the effect of subsequent UBC on the outcome of UTUC patients stratified by luminal–basal subtypes. The IHC expression of cytokeratin 5/6 (CK5/6), CK20, GATA3, and p53 was evaluated to assess relevant subtypes. Genetic characterization comprised TERTp, FGFR3, RAS, and TP53 status. Kaplan–Meier and Cox regression analyses were employed to assess the impact of clinicopathological variables and molecular profiles on progression-free survival (PFS) and overall survival (OS) in UTUC patients. The results indicated that no statistically significant differences were identified between the two groups concerning luminal–basal stratification and genetic analysis. In conclusion, bladder involvement is associated with shorter progression-free survival but does not appear to impact overall survival.

The third article, by Julius Drachneris et al., aims to address the need for more robust image-based predictive factors for the prediction of non-muscle-invasive papillary urothelial carcinoma (NMIPUC) recurrence. They analyzed a cohort of 157 patients treated with BCG. This analysis was conducted using features extracted from image patches sampled from whole-slide images of hematoxylin–eosin-stained transurethral resection (TUR) NPMIPUC specimens. They tested several variations of patch sampling and feature extraction networks to optimize model performance, and selected the model showing the best patient survival stratification for further testing in the context of clinical and pathological variables. The best-selected model revealed an independent prognostic value in the context of other clinical and pathologic variables (tumor stage, grade, and presence of tumor on the repeated TUR) with statistically significant patient risk stratification. In conclusion, the study suggests that MISL-based predictions can improve NMIPUC patient risk stratification.

The fourth article, by Francesco Pierconti et al., discusses the role of methylation analysis in non-muscle-invasive bladder cancer. A total of 782 patients with a diagnosis of non-muscle-invasive high-grade carcinoma (NMIBC) were evaluated. The Bladder EpiCheck test (BE) was performed in conjunction with cytology in all cases within 1 year after the completion of treatment. In 402 patients, the urinary samples were voided urine (UV), while in 380 cases, the samples were collected after bladder washing (IU). For all patients with invalid BE results, a second BE test was performed according to the instructions for use, which indicated that the test should be repeated with a new urinary sample if an invalid result was obtained. The final analysis revealed that the two different groups (UV and IU) had invalid BE results that appeared unrelated to the urinary samples. They summarized that, for patients for whom a BE test is planned, a combined approach of cytology and a methylation test is recommended.

The fifth article, by Yuji Nitta et al., analyzed the role of miR-138 and SOX9 in urothelial carcinoma. SOX9 was highly expressed in invasive urothelial carcinoma tissues. In contrast, miR-138 precursor transfection of T24 and UMUC2 cells significantly decreased SOX9 expression, indicating that SOX9 is a target of miR-138 in urothelial carcinoma. These findings suggest that miR-138–SOX9 signaling modulates the growth and invasive potential of urothelial carcinoma cells.

The article by Che-Yuan Hu et al. evaluated the role of Oct4 and Hypoxia Dual-Regulated Oncolytic Adenovirus armed with a shRNA-targeting dendritic cell immunoreceptor. They generated a Clec4a2 shRNA-expressing oncolytic adenovirus derived from Ad.LCY, designated Ad.shDCIR, aimed at inducing more robust antitumor immune responses. Their results show that treatment with Ad.shDCIR reduced Clec4a expression in DCs in cell culture. In conclusion, they suggest that Ad.shDCIR may be further explored as a combination therapy of virotherapy and immunotherapy for bladder cancer and likely other types of cancer.

The article by Konstantinos Kapriniotis et al. is a review of the literature regarding the prognostic role of circulating tumor DNA in the management of muscle-invasive bladder cancer. They showed first that detectable ctDNA levels before radical cystectomy are correlated with a higher risk of recurrence and worse overall prognosis after cystectomy. In addition, ctDNA status after NAC/neoadjuvant immunotherapy is predictive of the pathological response to these treatments, with persistently detectable ctDNA being associated with residual bladder tumor at cystectomy. Finally, detectable ctDNA levels post-cystectomy have been associated with disease relapse and worse disease-free survival (DFS) and overall survival (OS), and may identify a population with a survival benefit from adjuvant immunotherapy.

The last article, by Mohammad Jad Mousa, reviews the working definitions of ‘cisplatin ineligibility’ and ‘platinum ineligibility’ in mUC clinical trials and the standard of care in both categories. Then, they review select clinical trials for the frontline treatment of cisplatin- and platinum-ineligible mUC patients on ClinicalTrials.gov. They rethink the value of classifying patients by cisplatin or platinum ineligibility in the frontline setting in the post-EVP era, and lastly they discuss new priority goals to tailor predictive, monitoring, and prognostic biomarkers to these emergent therapies.

## 3. Conclusions

This collection of articles focuses on the significance of biomarkers in the diagnosis, prognosis, and treatment of urothelial carcinoma (UC). Although significant advances have been achieved, many questions still exist, necessitating further validation and standardization. Prospective trials will be crucial in incorporating these biomolecular findings into patient care practices. Additionally, tackling health disparities and ensuring equal access to advanced diagnostic tools must remain a top priority as we move forward.

We appreciate the valuable reviews, original data, and innovative perspectives of these contributors. This Special Issue highlights the growing importance of biomarkers in urothelial carcinoma, laying the groundwork for future innovations that will significantly impact patient outcomes.
